# Claudin 1 Mediates TNFα-Induced Gene Expression and Cell Migration in Human Lung Carcinoma Cells

**DOI:** 10.1371/journal.pone.0038049

**Published:** 2012-05-31

**Authors:** Atsushi Shiozaki, Xiao-hui Bai, Grace Shen-Tu, Serisha Moodley, Hiroki Takeshita, Shan-Yu Fung, Yingchun Wang, Shaf Keshavjee, Mingyao Liu

**Affiliations:** 1 Latner Thoracic Surgery Research Laboratories, University Health Network Toronto General Research Institute, Toronto, Canada; 2 Department of Surgery, Faculty of Medicine, University of Toronto, Toronto, Canada; Cincinnati Children's Hospital Medical Center, United States of America

## Abstract

Epithelial-mesenchymal transition (EMT) is an important mechanism in carcinogenesis. To determine the mechanisms that are involved in the regulation of EMT, it is crucial to develop new biomarkers and therapeutic targets towards cancers. In this study, when TGFβ1 and TNFα were used to induce EMT in human lung carcinoma A549 cells, we found an increase in an epithelial cell tight junction marker, Claudin 1. We further identified that it was the TNFα and not the TGFβ1 that induced the fibroblast-like morphology changes. TNFα also caused the increase in Claudin-1 gene expression and protein levels in Triton X-100 soluble cytoplasm fraction. Down-regulation of Claudin-1, using small interfering RNA (siRNA), inhibited 75% of TNFα-induced gene expression changes. Claudin-1 siRNA effectively blocked TNFα-induced molecular functional networks related to inflammation and cell movement. Claudin-1 siRNA was able to significantly reduce TNF-enhanced cell migration and fibroblast-like morphology. Furthermore, over expression of Claudin 1 with a Claudin 1-pcDNA3.1/V5-His vector enhanced cell migration. In conclusion, these observations indicate that Claudin 1 acts as a critical signal mediator in TNFα-induced gene expression and cell migration in human lung cancer cells. Further analyses of these cellular processes may be helpful in developing novel therapeutic strategies.

## Introduction

Inflammatory mediators are important constituents of local environment for tumors, and evidences suggest that they are closely linked to cancer and inflammation [1]. For instance, many chronic inflammatory diseases are associated with greater risk of cancer [2]. One of the key mediators implicated in inflammation-associated cancer is tumor necrosis factor alpha (TNFα) [3]. Although TNFα was first identified for its ability to induce rapid hemorrhagic necrosis of experimental cancers, TNFα is now known to be produced in cancer cells as an endogenous tumor promoter [2], [3]. Animal model studies demonstrate that TNFα has pro-cancer actions [4], [5]. TNFα−/− and TNFR1−/− mice are resistant to chemically induced carcinogenesis in the skin [6]. TNFR1−/− mice are resistant to chemical carcinogenesis in the liver [7], and in the development of liver metastasis in experimental colon cancer [8]. Furthermore, TNFα is frequently detected in human cancers with poor prognosis, such as ovarian, renal and breast cancers [9]. TNFα has been suggested as a target for renal-cell carcinoma treatment [10].

TNFα is involved in epithelial-mesenchymal transition (EMT) [11]. It enhances transforming-growth factor β1 (TGFβ1)-induced EMT in multiple cancer cell types [12], [13]. TNFα induces the malignant progression of epithelial tumors by controlling cell migration, invasion and metastasis. During the progression of EMT, tight junction (TJ) proteins, such as Claudins and Occludins, and adherens junction proteins, such as E-Cadherin, are usually down-regulated [12]–[17]. TNFα also induces internalization of TJ proteins [18], decreases trans-epithelial electrical resistance, and increases the paracellular permeability of ions and normally impermeable molecules [19].

The Claudin family of proteins consists of 24 members and plays an integral role in the formation and function of tight junctions [20], [21]. Claudin family members interact with each other through homo- and heterophilic interactions [21], [22]. As TJ proteins, Claudins are crucial for the maintenance of cellular polarity and paracellular transportation of molecules. Claudin proteins can be up-regulated and mis-localized in cancer cells [21]. The expression of Claudin 1 increases during tumorigenesis of colon cancer [23], melanoma [24], oral squamous cell carcinoma [25] and hepatocellular carcinoma [26].

In the present study, human lung carcinoma A549 cells were treated with TNFα and TGFβ1 to induce EMT. The expression of Claudin 1 was increased in response to TNFα challenge. Further studies indicated that Claudin 1 plays a crucial role in TNFα-induced gene expression and cell migration in human lung carcinoma cells.

## Materials and Methods

### Cell line, antibodies and other reagents

**Figure 1:**
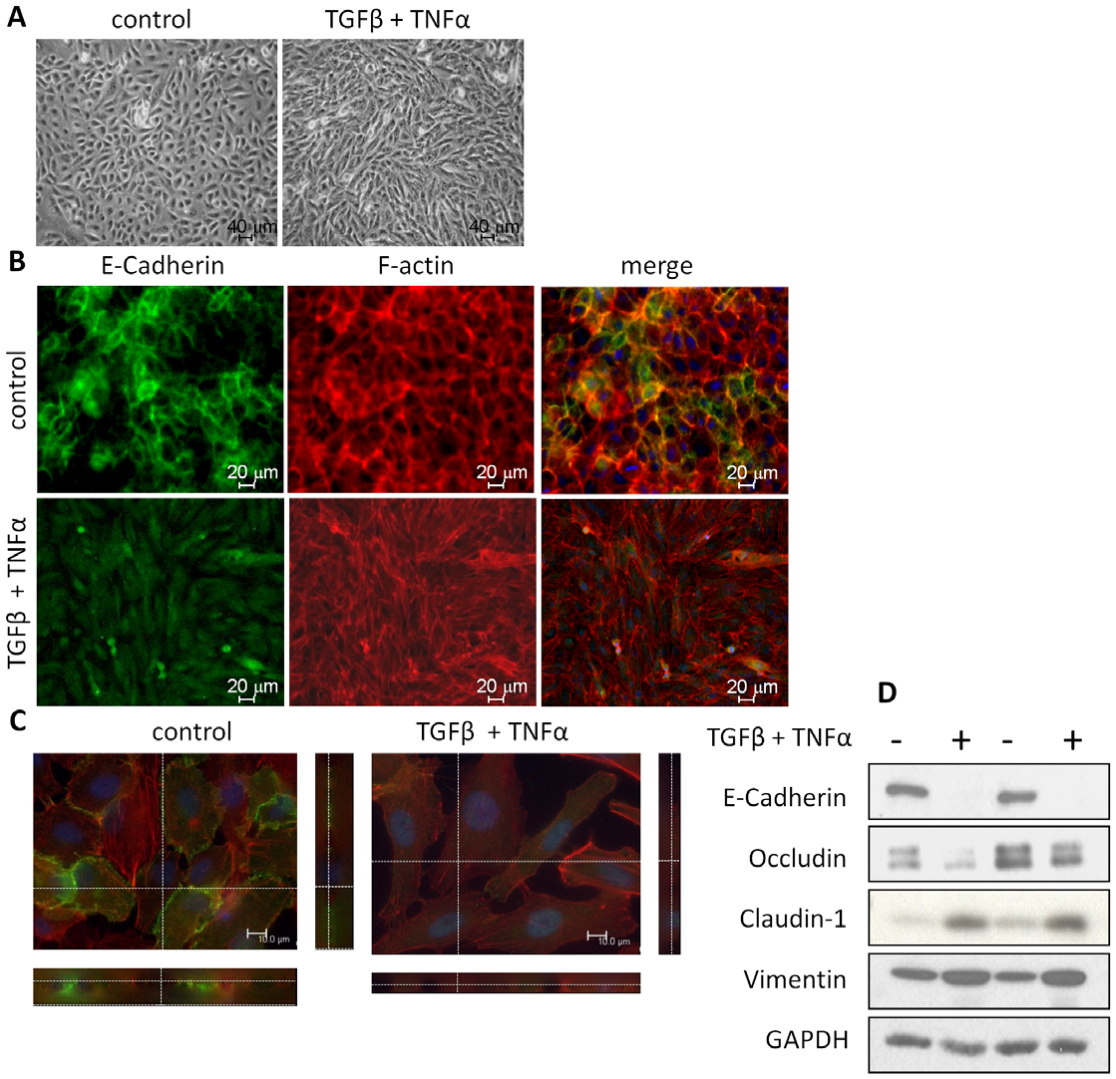
TNFα and TGFβ1 induce EMT in A549 cells. (A) The combined treatment of TNFα (20 ng/ml) and TGFβ1 (10 ng/ml) for 72 h induced morphological alterations characterized as fibroblast-like cells. (B) TNFα and TGFβ1 treatment reduced expression of E-Cadherin at cell-to-cell contacts, and increased formation of F-actin stress fibers. Cells were immunostained with an anti-E-Cadherin antibody and counterstained F-actin and nuclei with rhodamine phalloidin and Hoechst 33342, respectively. (C) The redistribution of E-cadherin after TGFβ and TNFα treatment from the cell-to-cell contacts to cytosol was further demonstrated with confocal microscopy at higher maginification. (D) The 72 h treatment with TNFα and TGFβ1 decreased expressions of E-Cadherin and Occludin, epithelial markers, and increased expressions of Vimentin, a mesenchymal marker, in A549 cells. Surprisingly, the expression of Claudin 1, an epithelial marker, was increased as analyzed by western blotting.

**Figure 2:**
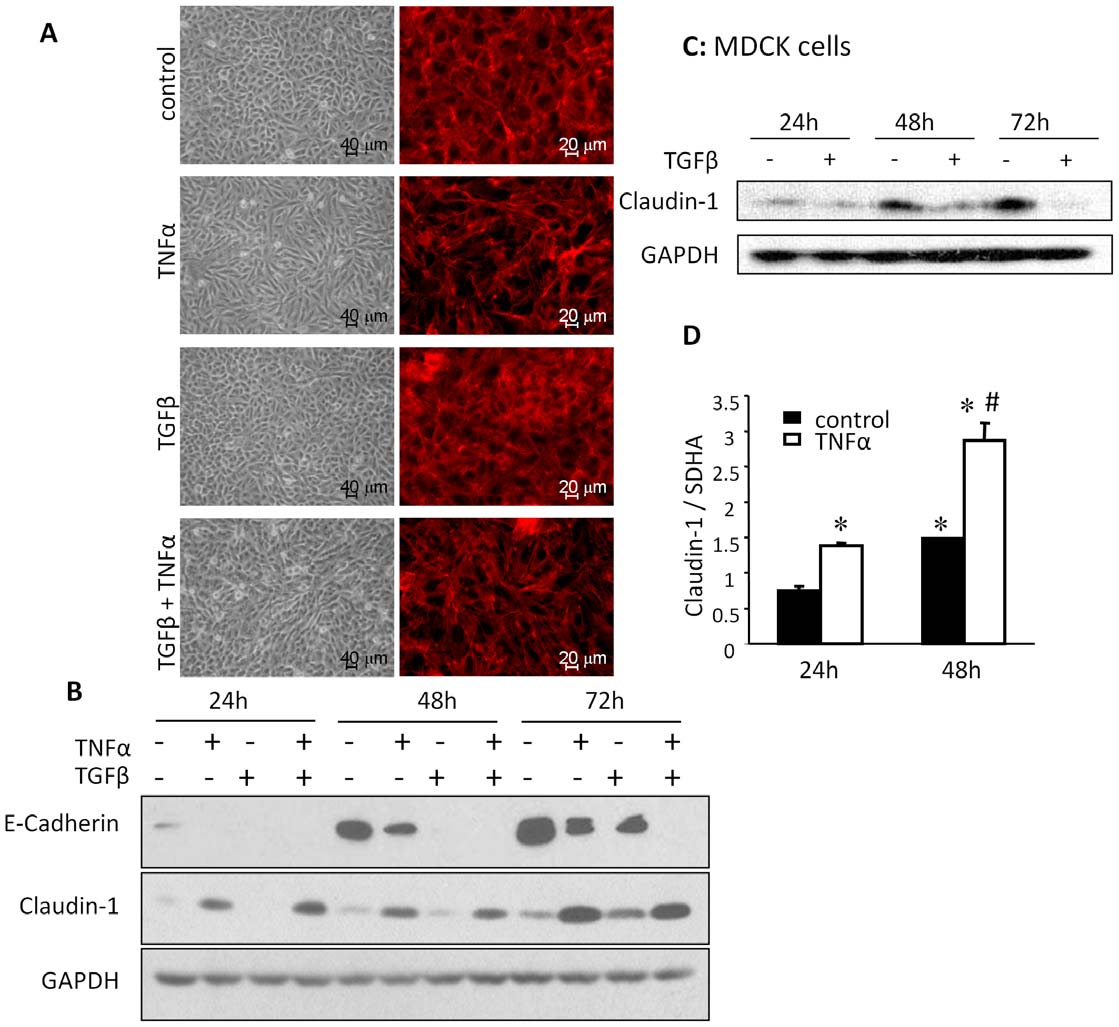
TNFα alone induces fibroblast-like morphology and Claudin 1 expression. (A) TNFα alone induced fibroblast-like morphological alteration, reduced cell-to-cell contacts, and increased F-actin stress fibers. A549 cells treated with TNFα, TGFβ1 or TNFα and TGFβ1 together for 72 h were examined with light microscopy and stained with rhodamine phalloidin to visualize F-actin structures. TNFα-induced changes were similar as that of the TNFα and TGFβ1 treatment, whereas TGFβ1 alone did not induce these changes. (B) Protein levels of E-Cadherin were more effectively reduced by TGFβ1 than TNFα. TNFα alone increased Claudin 1 expression, whereas TGFβ1 had little effects on Claudin 1 after 24 h or 48 h treatment, and even increased it after 72 h treatment, as determined by western blotting. (C) In MDCK cells, TGFβ1 reduced Claudin 1 after 24, 48 or 72 h treatment. (D) Expression levels of Claudin 1 mRNA were significantly increased by TNFα in a time-dependent manner as measured by real-time quantitative RT-PCR. Mean ± SEM. *n* = 3 experiments. *p<0.05 (compared with control at 24 h). #*p*<0.05 (compared with control at 48 h).

**Figure 3:**
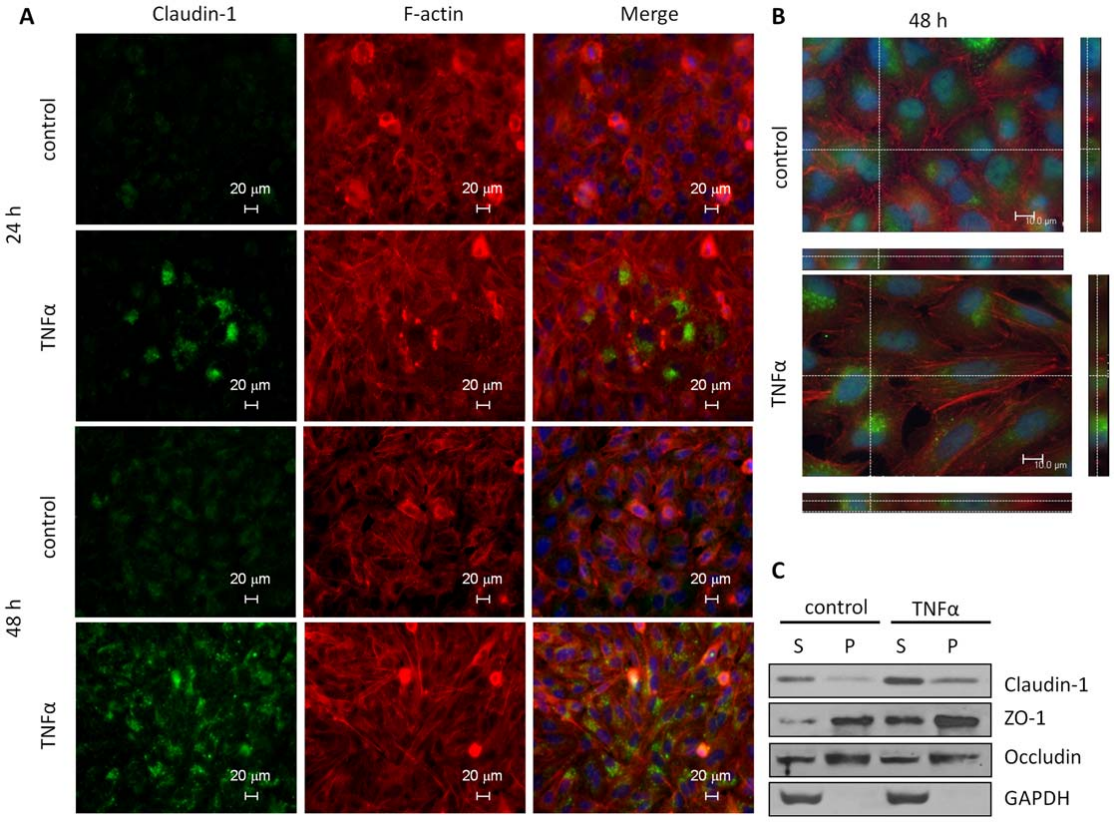
TNFα-induced Claudin 1 protein is mainly in the cytosolic fraction. (A) At 24, 48 h after treating the cells with TNFα, expression of Claudin 1 protein was increased mainly in cytoplasm. A549 cells were immunostained with an anti-Claudin 1 antibody and counterstained F-actin and nuclei with rhodamine phalloidin and Hoechst 33342, respectively. (B) Confocal microscopy further confirmed the cytosolic distribution of Claudin 1 in both control and TNFα groups. (C) Claudin 1 and GAPDH were mainly found in the Triton X-100 soluble (S) cytoplasm fraction, whereas ZO-1 and Occludin were mainly in the Triton X-100 insoluble cytoskeletal pellets (P). A549 cells treated with or without TNFα (20 ng/ml for 24 h) were lyzed and separated into Triton X-100 soluble and insoluble fractions for immunoblotting.

**Figure 4:**
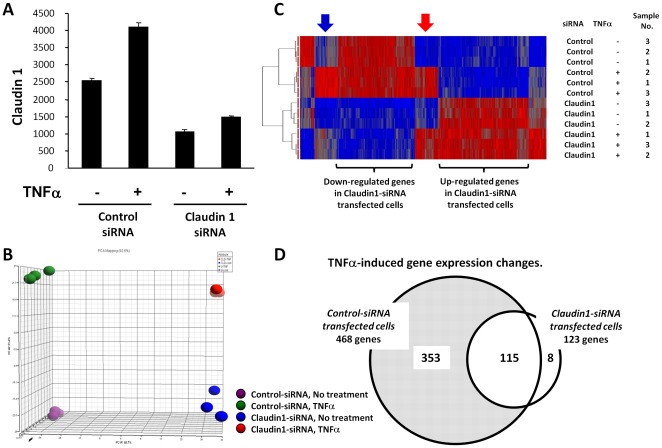
Down-regulation of Claudin 1 with siRNA significantly blocked TNFα-induced gene expression. (A) Claudin 1 siRNA effectively reduced both basal and TNFα-induced gene expression of Claudin 1 (reading from microarray). (B) Principle Component Analysis (PCA) showed that the overall gene expression profiles are separated based on the Claudin 1 siRNA transfection and TNFα treatment. (C) Hierarchical clustering analysis demonstrates that gene expression patterns are highly dependent upon Claudin 1 siRNA transfection and TNFα treatments. A two-way ANOVA showed that 2,490 genes were significantly different. Red: up-regulated; blue: down-regulated. (D) Claudin 1 siRNA blocked 75% of the TNFα-induced gene expression changes. The gene expressions that were significantly changed by TNFα were defined with FDR q value less than 5.0% and fold of change greater than 1.3 by SAM analysis. Venn diagram shows that 75% of TNFα-induced expression changes were not shown in Claudin 1 siRNA transfected cells.

**Figure 5:**
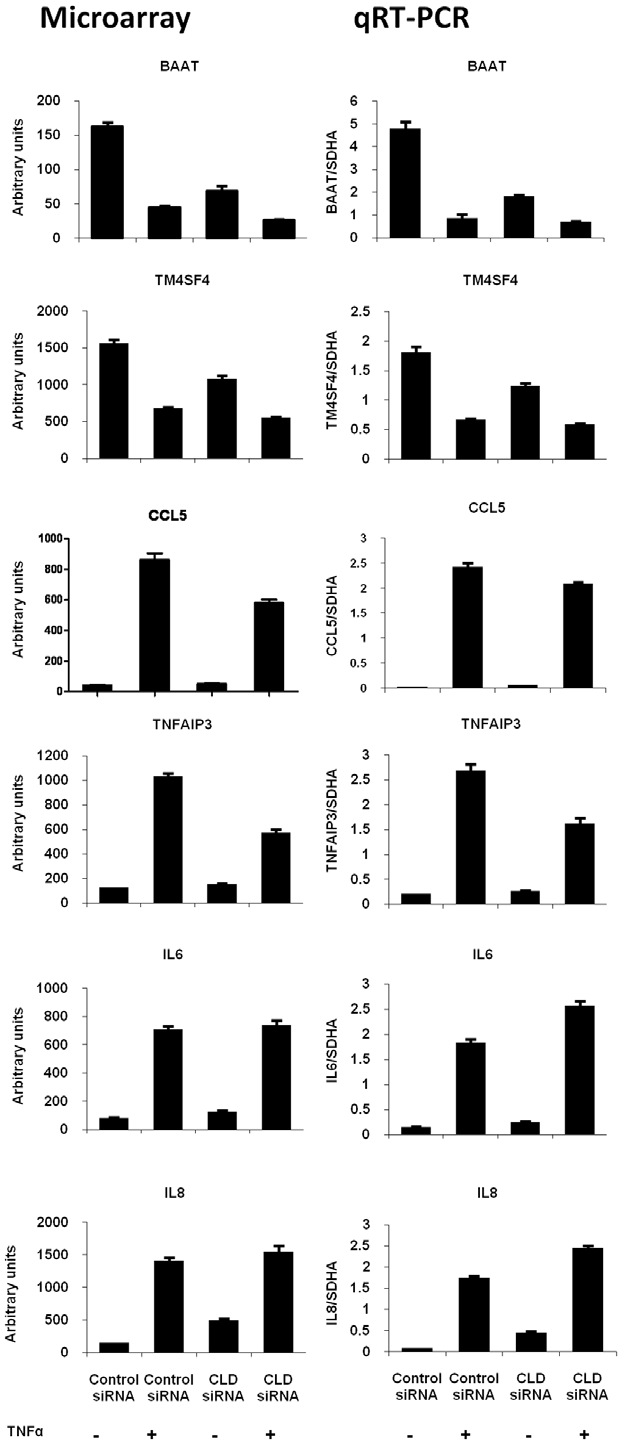
Validation of gene expression by real-time quantitative RT-PCR. The expression of six genes (BAAT, TM4SF4, CCL5, TNFAIP3, IL-6, and IL-8) in Claudin 1 siRNA transfected cells were compared with control siRNA transfected cells treated with or without TNFα. The microarray results are plotted in the left column. The qRT-PCR results normalized to the level of SDHA are plotted in the right column. N = 4, Mean ± SEM.

**Table 1:** Top 20 up-regulated genes induced by TNFα in Control-siRNA or Claudin 1-siRNA transfected A549 cells.

Up-Regulated Genes						
Gene Symbol	Gene Name	Gene ID	Control-siRNA		Claudin 1-siRNA	
			Fold Change	q-value (%)	Fold Change	q-value (%)
CCL5	chemokine (C-C motif) ligand 5	NM_002985	21.729	<0.001	11.471	<0.001
CLEC4E	C-type lectin domain family 4, member E	NM_014358	14.712	<0.001	6.523	<0.001
IL8	interleukin 8	NM_000584	9.634	<0.001	3.136	<0.001
IL6	interleukin 6 (interferon, beta 2)	NM_000600	8.530	<0.001	5.958	<0.001
TNFAIP3	tumor necrosis factor, alpha-induced protein 3	NM_006290	8.188	<0.001	3.764	<0.001
CCL2	chemokine (C-C motif) ligand 2	NM_002982	4.466	<0.001	2.387	<0.001
SOD2	superoxide dismutase 2, mitochondrial	NM_001024465	4.404	<0.001	2.617	<0.001
IL1A	interleukin 1, alpha	NM_000575	4.258	<0.001	4.128	<0.001
CFB	complement factor B	NM_001710	4.202	<0.001	2.020	<0.001
IFI44	interferon-induced protein 44	NM_006417	3.833	<0.001	2.448	3.500
EFNA1	ephrin-A1	NM_004428	3.819	<0.001	1.718	≥5.0
PTX3	pentraxin-related gene, rapidly induced by IL-1 beta	NM_002852	3.762	<0.001	1.982	4.154
ASS1	argininosuccinate synthetase 1	NM_000050	3.589	<0.001	2.003	<0.001
PTPLAD2	protein tyrosine phosphatase-like A domain containing 2	NM_001010915	3.432	<0.001	1.988	<0.001
VCAM1	vascular cell adhesion molecule 1	NM_001078	3.418	<0.001	1.961	4.221
GPR141	G protein-coupled receptor 141	NM_181791	3.343	<0.001	2.805	<0.001
C15orf48	chromosome 15 open reading frame 48	NM_032413	3.239	<0.001	2.476	<0.001
LAMC2	laminin, gamma 2	NM_005562	3.082	<0.001	2.696	<0.001
IFIT1	interferon-induced protein with tetratricopeptide repeats 1	NM_001548	3.002	<0.001	2.240	≥5.0
TNFRSF9	tumor necrosis factor receptor superfamily, member 9	NM_001561	2.984	<0.001	2.721	<0.001

**Table 2:** Top 10 down-regulated genes induced by TNFα in Control-siRNA or Claudin 1-siRNA transfected A549 cells.

Down-Regulated Genes						
Gene Symbol	Gene Name	Gene ID	Control-siRNA		Claudin 1-siRNA	
			Fold Change	q-value (%)	Fold Change	q-value (%)
BAAT	bile acid Coenzyme A: amino acid N-acyltransferase (glycine N-choloyltransferase)	NM_001701	−3.632	<0.001	−2.580	≥5.0
TM4SF4	transmembrane 4 L six family member 4	NM_004617	−2.289	0.387	−1.943	≥5.0
HLA-DMB	major histocompatibility complex, class II, DM beta	NM_002118	−1.963	2.306	−1.985	≥5.0
OLFML3	olfactomedin-like 3	NM_020190	−1.865	3.152	−1.623	≥5.0
C12orf27	chromosome 12 open reading frame 27	ENST00000315185	−1.822	3.186	−1.579	≥5.0
METTL7A	methyltransferase like 7A	NM_014033	−1.733	3.818	−1.324	≥5.0
C5	complement component 5	NM_001735	−1.644	3.818	−1.521	≥5.0
ACSM3	acyl-CoA synthetase medium-chain family member 3	NM_005622	−1.630	3.152	−1.651	≥5.0
TRIML2	tripartite motif family-like 2	NM_173553	−1.626	3.152	−1.440	≥5.0
SEMA3E	sema domain, immunoglobulin domain (Ig), short basic domain	NM_012431	−1.609	3.152	−1.262	≥5.0

**Figure 6:**
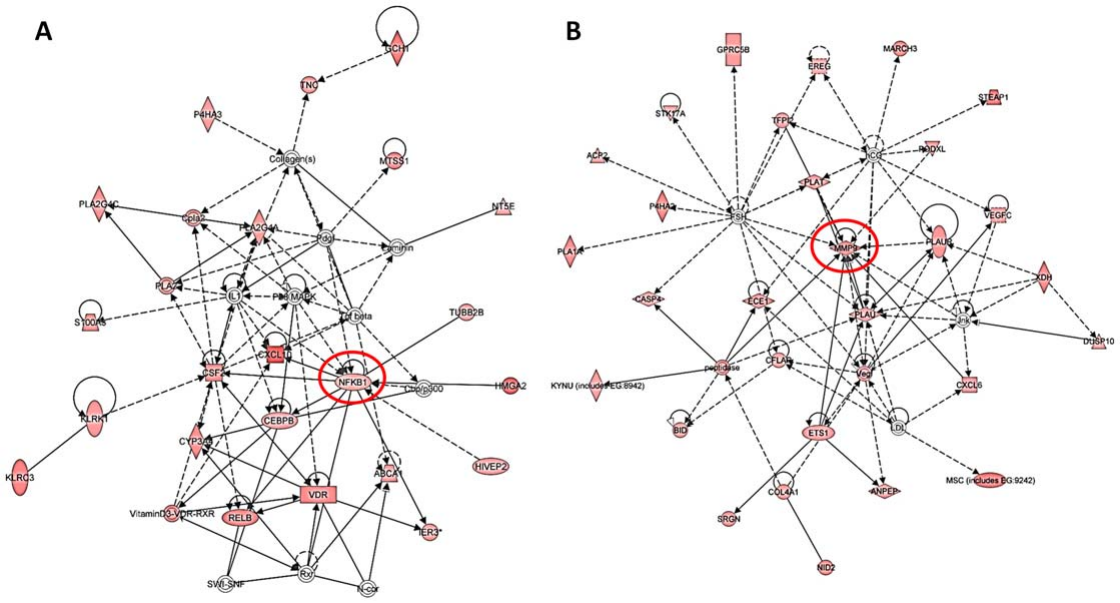
Knocking down Claudin 1 with siRNA reduced gene expression related to inflammation and cell migration. Ingenuity Pathway Analysis was performed on 353 genes which were significantly changed by TNFα treatment in Control siRNA transfected cells, but not in Claudin 1 siRNA transfected cells (highlighted area with dots on Venn diagram in Fig. 4D). (A) Signal network related to inflammation. Note that NFκB is located at the center of the network. (B) Signal network related to cell movement. Note MMP-9 is located at the center of the network.

**Table 3:** Top Bio Functions of TNFα-Induced Genes Blocked by Claudin 1 siRNA, as analyzed by Ingenuity Pathway analysis.

Top Bio Functions		
Molecular and Cellular Functions		
Name	p-value	Number of Molecules
Antigen Presentation	8.33E-12–6.75E-03	74
Cellular Development	6.06E-09–6.75E-03	62
Gene Expression	2.94E-07–7.93E-03	21
Cellular Movement	2.16E-06–7.36E-03	53
Cell Death	2.90E-06–7.41E-03	86

**Figure 7:**
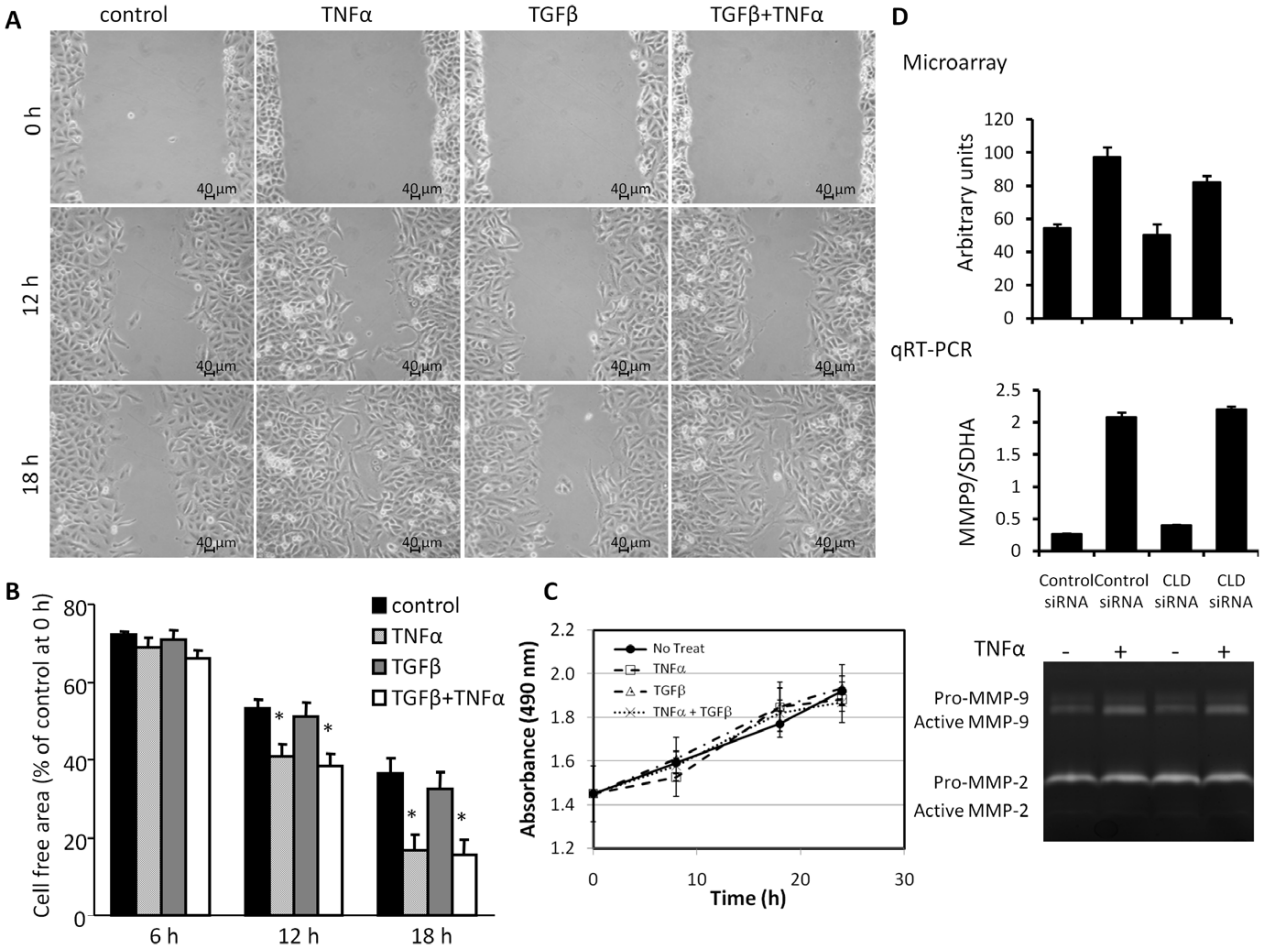
TNFα promotes migration of A549 cells. (A) TNFα enhanced cell migration in a wound healing assay. After mechanical wounding, confluent A549 cells were treated with TNFα (20 ng/ml), TGFβ1 (10 ng/ml) or TNFα and TGFβ1 together. Representative photomicrographs of the wounded cell monolayer are shown. (B) Percentage of cell free area in each condition was calculated. *n* = 4. Mean ± SEM. *p<0.05 (compared with control at the same time point). (C) TNFα and/or TGFβ1 did not affect cell proliferation determined by MTS assay. (D) TNFα treatment for 24 h increased the gene expression of MMP-9 (as determined by microarray and qRT-PCR) and the level of active MMP-9, while the levels of both pro- and active-MMP 2 had no dramatic changes, as determined by gelatin zymography assay.

**Figure 8:**
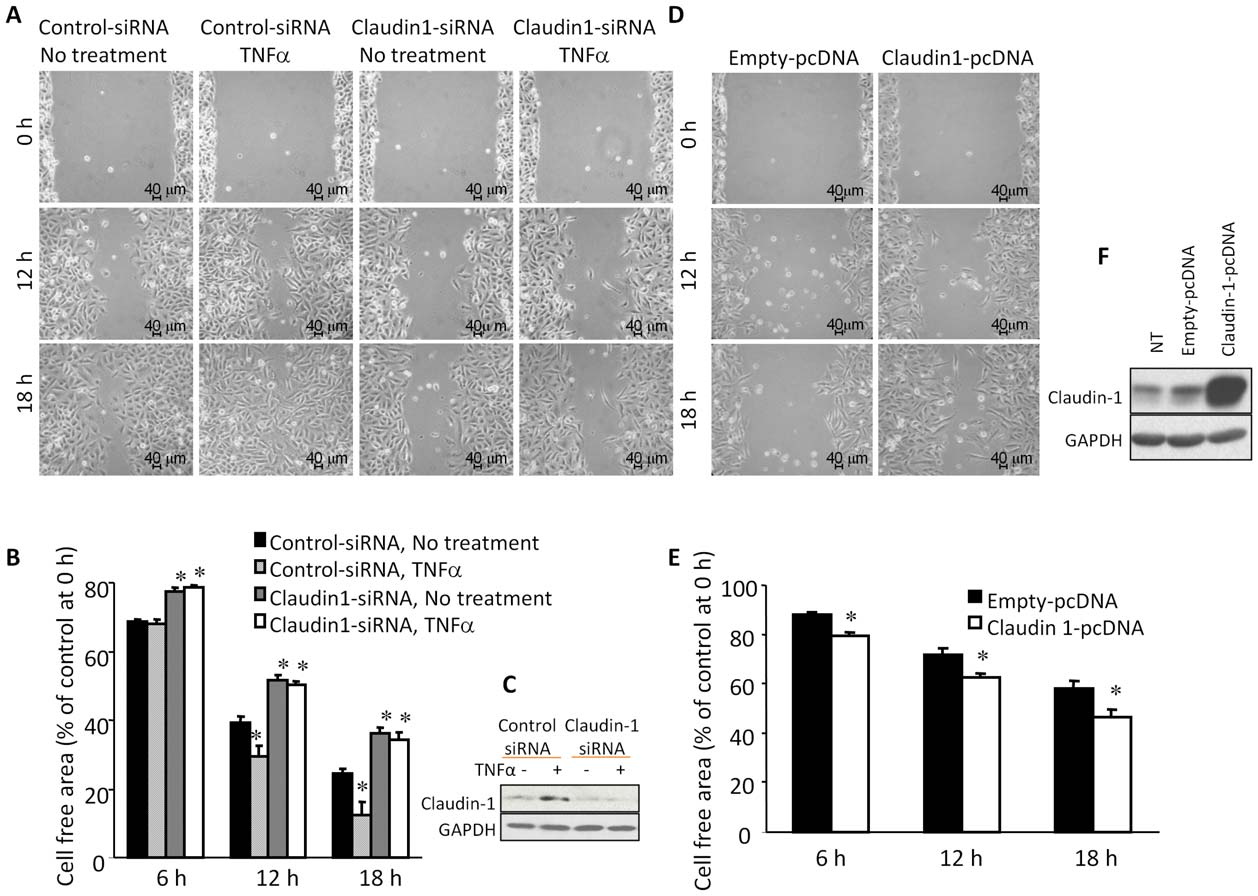
Claudin 1 expression levels affect cell migration. (A) Down regulation of Claudin 1 with siRNA reduced spontaneous as well as TNFα-enhanced migration of A549 cells. The control or Claudin 1 siRNA transfected A549 cells were cultured until confluent, mechanically wounded, and then treated with or without 20 ng/ml TNFα. Representative photomicrographs of wounded cell monolayer are shown. (B) Percentage of cell free area in each condition was calculated. *n* = 4. Mean ± SEM. *p<0.05 (compared with control siRNA transfected cells at the same time point). (C) Claudin 1 siRNA effectively reduced both basal and TNFα induced expression levels of Claudin 1 in A549 cells. Control or Claudin 1 siRNA transfected cells were treated with or without TNFα (20 ng/ml for 24 h) and harvested for western blotting. (D) Over-expression of Claudin 1 enhanced cell migration. (E) Percentage of cell free area in each condition was calculated. *n* = 4. Mean ± SEM. *p<0.05, compared with empty vector transfected cells at the same time point. (F) Claudin 1-pcDNA effectively increased the expression level of Claudin 1. A549 cells were transfected with empty vector or Claudin 1-pcDNA for 24 h, and harvested for western blotting.

**Figure 9:**
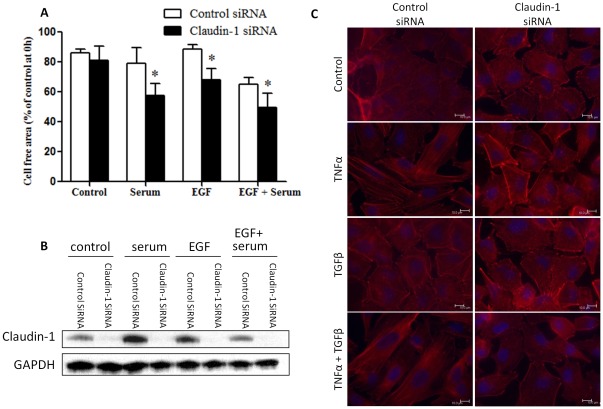
Reducing Claudin 1 protein levels enhanced serum and/or EGF induced A549 cell migration, and Claudin 1 siRNA reduced TNFα-induced morphological changes of A549 cells. Cells were transfected with Claudin 1 or control siRNA. The confluence monolayers were serum starved, mechanically wounded and then stimulated with serum (10% FBS) and/or EGF (50 ng/ml). The wounded areas at 12 h were quantified. N = 4, Mean ± SEM. *P<0.05 vs. control siRNA treated group. (B). Claudin 1 siRNA clearly reduced Claudin 1 protein levels as shown by Western blotting. (C). Claudin 1 siRNA reduced TNFα- and TNFα plus TGFβ-induced morphological changes as shown by F-actin staining at 48 h. Similar results were also found after 24 h or 72 h of TNFα treatment.

Human lung adenocarcinoma A549 cells and MDCK cells were grown in DMEM medium supplied with 10% fetal bovine serum, 1% penicillin-streptomycin, and 1% glutamine. Cells were cultured in a standard humidified incubator at 37°C with 5% CO_2_.

Antibodies for ZO-1, E-Cadherin, Occludin and Claudin-1 were from Zymed Laboratories (S. San Francisco, CA). Antibody for Vimentin was from Cell Signaling Technology (Beverly, MA). Antibody for GAPDH was from Santa Cruz Biotechnology (Santa Cruz, CA). Horseradish peroxidase (HRP)-conjugated goat anti-mouse and anti-rabbit secondary antibodies were from Amersham Pharmacia Biotech (Piscataway, NJ). Alexa Fluor 488 labeled goat anti-mouse and anti-rabbit secondary antibodies, rhodamine phalloidin, and Hoechst dye 33342 were from Invitrogen (Carlsbad, CA). Human TNFα was from R&D Systems Inc. (Minneapolis, MN). Human TGFβ1 was from Austral Biological (San Ramon, CA).

### Immunofluorescent staining and microscopy

Cells were stained as previously described [27]–[29]. A549 cells were cultured on glass coverslips (VWR, Mississauga, Canada). After different treatments, cells were fixed with 4% paraformaldehyde, permeabilized with 0.1% Triton X-100, blocked with 1% BSA, stained with designated antibodies, rhodamine phalloidin and Hoechst dye 33342. After gentle washing, coverslips were mounted on glass slides with Dako fluorescence mounting medium (Dako, Mississauga, Canada). Slides were examined as previously described [27]–[29]. Isotype-matched mouse or rabbit IgG were used as negative control with the same dilutions as the primary antibodies.

### Protein studies

Immunoblotting experiments were performed according to procedures described previously [30]–[33]. Triton X-100 soluble and insoluble protein fractions were prepared as described by Nishiyama and coworkers [34]. Briefly, cells were lysed with modified radioimmune precipitation assay buffer containing 1% Triton X-100. Cell lysate was centrifuged (12,000 rpm for 10 min at 4°C), and the supernatant was collected as the Trinton X-soluble fraction. The remaining pellet was resuspended in 60 µl of lysis buffer containing 1% SDS. The resulting suspension was centrifuged (12,000 rpm for 10 min at 4°C) and the supernatant was collected as the Triton-X insoluble fraction.

### Real-time quantitative RT-PCR

The qRT-PCR primers used for human Claudin-1 were 5′-GCGCGATATTTCTTCTTGCAGG-3′ (Forward) and 5′-TTCGTACCTGGCATTGACTGG-3′ (Reverse) [35]. The primers used for human succinate dehydrogenase complex subunit A (SDHA) were 5′-CGGCATTCCCACCAACTAC-3′ (Forward) and 5′-GGCCGGGCACAATCTG-3′ (Reverse) [36]. Primers for other genes are available upon request. Total RNA was extracted from A549 cells with TRIZOL Reagent (Invitrogen). qRT-PCR was performed using 2×QuantiTect SYBR Green PCR kit (Qiagen, Mississauga, Canada) on LightCycler480 (Roche, Mannheim, Germany) as described [37], [38]. Each assay included a standard curve of five serial dilutions and a no-template negative control. The gene expression levels were normalized to the level of SDHA as a house-keeping gene.

### siRNA transfection

Claudin 1 siRNA was purchased from Santa Cruz Biotechnology. Cells were transfected with 50 nM Claudin 1 siRNAs using the oligofectamine reagent (Invitrogen) [31]–[33], [36], [39]. The medium containing siRNA was replaced with a fresh medium with or without TNFα 24 h after transfection. The siSTABLE V2 non-targeting siRNA#1 from Dharmacon (Lafayette, CO) was used as a negative control. For protein studies, the siRNA transfected cells were harvested at different days after transfection.

### Microarray and data analysis

For microarray study, four groups were prepared (i.e., cells treated with or without TNFα and in the presence of Claudin 1 siRNA or control siRNA) and were tested with three biological replicates for each group. Total RNA was extracted using RNeasy kit (Qiagen, Valencia, CA), and cDNA was synthesized with High-Capacity cDNA Reverse Transcription kits (Applied Biosystems, Foster City, CA) on a PTC-100™ Programmable Thermal controller (MJ Research Inc., Watertown, MA). The RNA Integrity Number (RIN) was determined by Agilent Bioanalyzer 2100 (Agilent Technologies, Inc., Santa Clara, CA). Human Gene ST 1.0 chips (28,132 spotted genes) from Affymetrix (Santa Clara, CA) were used. Affymetrix CEL files were imported into Partek software (Partek Inc., St. Louis, MO) using the default Partek normalization parameters. Probe-level data were pre-processed with robust multi-array average (RMA) analyses, which include background correction, normalization, and summarization. Data normalization was performed across all arrays, using quartile normalization. The processed values were then compiled, or summarized, using the median polish technique, to generate a single measure of expression. Principle Component Analysis (PCA) was performed using Partek. Hierarchical cluster analysis was performed with significantly changed genes (p<0.001) using two-way ANOVA. Differential expression analysis was performed using Significance Analysis of Microarray (SAM) [40]. Signal transduction network was analyzed with Ingenuity Pathway Analysis (IPA)(Ingenuity Systems, Inc., Redwood City, CA) [36]. The original microarray data have been deposited to GEO Repository (accession number GSE 32254).

### Wound-healing assay

Wounds were created in confluent cells using a pipette tip [28]. The cells were then rinsed with medium to remove floating cells and debris. TNFα and/or TGFβ1 containing medium was added. To test the effects of serum and/or EGF on cell migration, after siRNA transfection confluent cells were serum starved before wounding. Cells were then treated with 10% FBS, and/or 50 ng/ml EGF [41]. The culture plates were incubated at 37°C. Wounds were measured at 0, 6, 12 and 18 h. Assays were repeated four times for each condition.

### Cell proliferation assay

The proliferation assays were performed to determine the effect of TNFα and/or TGFβ1 on the cell growth over time as described previously [42]. In brief, A549 cells were seeded into 96-well cell culture plates at a density of 5×10^3^ cells/well and incubated for 48 h prior to the treatments. The medium was replaced with 100 µl fresh one containing 20 ng/ml TNFα and/or 10 ng/ml TGFβ1. The assay was performed at 0, 8, 18 and 24 h after the treatments in quadruplicates with CellTiter 96^R^ AQueous One Solution Cell Proliferation Assay (Promega, Madison, WI). At each time point, 20 µl of the assay solution was added to each well and incubated for 1 h at 37°C. The plate was then read on a 96 well plate reader (ThermoLab system, Opsys MR) at 490 nm. The absorbance values were plotted as a function of time for each treatment to show the cell proliferation profile.

### Gelatin zymography

The cell-conditioned media were diluted with sample buffer (5% SDS, 20% glycerol in 0.5 M Tris, pH 6.8, containing 0.02% bromophenol blue), and loaded in a 10% zymogram gel containing 0.1% gelatin (Sigma-Aldrich, St. Louis, MO) [27]. After electrophoresis, the gels were washed for 30 min in 2.5% Triton X-100 and incubated overnight at 37°C in 50 mM Tris, pH 7.4, 5 mM CaCl_2_, 0.02% Brij 35. The gels were stained with 0.5% Coomassie Blue R-250 in 50% methanol and 10% acetic acid overnight at room temperature on a rotary shaker. The gels were de-stained for 5 h in 50% methanol and 10% acetic acid. The areas where the staining was digested were identified.

### Claudin 1-pcDNA transfection

Claudin 1-pcDNA3.1/V5-His plasmid was given by Dr. Patricia Pintor dos Reis and Dr. Suzanne Kamel-Reid (University of Toronto), and an empty-pcDNA3.1/V5-His plasmid was used as a negative control [25]. Cells were seeded in a 6-well plate at 5.0×10^5^ cells/well and incubated overnight at 37°C. Cells were transfected with 500 ng/well Claudin 1-pcDNA or empty-pcDNA plasmids using Lipofectamine™ 2000 reagent (Invitrogen). The media containing plasmids were replaced with fresh medium with or without TNFα at 24 h after transfection. For protein studies, the siRNA transfected cells were harvested at 48 h after transfection.

### Statistical analysis

Statistical analyses were carried out using Tukey-Kramer HSD test. Differences are considered significant when the P value was less than 0.05. Statistical analyses were performed using JMP version 5 (SAS Institute Inc., Cary, NC).

## Results

### Induction of EMT by TGFβ1 and TNFα in human lung carcinoma A549 cells

To investigate EMT in human lung carcinoma A549 cells, we treated cells with TNFα (20 ng/ml) and TGFβ1 (10 ng/ml) for 72 h. The combined treatment of TNFα and TGFβ1 induced morphological changes characterized as fibroblast-like cells (Figure 1A). Immunofluorescent staining demonstrated that the combined treatment with TNFα and TGFβ1 reduced expression of E-Cadherin, and re-distributed E-Cadherin from surface membrane to more diffuse in the cytoplasm (Figure 1B left panel). These changes were confirmed with confocal microscopy at higher magnification (Figure 1C). The F-actin staining showed strong cortical staining in the control cells, whereas more stress fibers can be seen in elongated fibroblast-like cells after TGFβ1 and TNFα treatment (Figure 1B, middle panel). Western blotting revealed that TNFα and TGFβ1 together decreased expressions of E-Cadherin and Occludin, typical epithelial adherens and TJ marker proteins, and increased expressions of Vimentin, a typical mesenchymal marker (Figure 1D). Claudin 1, one of the epithelial TJ markers, however, was increased after TGFβ1 and TNFα treatment (Figure 1D).

### TNFα induces morphologic alteration and Claudin 1 expression in A549 cells

To investigate the main signal that induces the morphologic alternation, we treated A549 cells with TNFα and/or TGFβ1 for 72 h. The changes in cell morphology and F-actin structures were determined with phase contrast microscopy and F-actin staining. TNFα alone induced the fibroblast-like morphological change of the cells with formation of F-actin stress fibers, and reduced cell-to-cell contact. On the other hand, TGFβ1 alone did not induce these changes (Figure 2A).

To investigate whether the change in the morphology of the cells are associated with changes of EMT markers, we treated cells with TNFα, TGFβ1, or the both. In untreated cells, the protein levels of E-Cadherin continued to increase from 24 h to 72 h. In TGFβ1 treated cells, E-Cadherin levels were inhibited; the band was seen only at the 72 h. In TNFα treated cells, E-Cadherin protein was found at 48 h and 72 h groups at lower levels than untreated controls. The inhibitory effect was enhanced by the combined TNFα and TGFβ1 treatment (Figure 2B). Claudin 1 expression was increased by TNFα alone. TGFβ1 alone slightly reduced Claudin 1 at 24 h and 48 h. The effects of the combined use of TNFα and TGFβ1 on Claudin 1 levels were very similar to the effect of TNFα alone (Figure 2B). In MDCK cells, TGFβ1 decreased Claudin 1 expression in a time dependent fashion [15]. When we stimulated MDCK cells with TGFβ1 (10 ng/ml), decreased Claudin 1 was also found (Figure 2C), suggesting the less inhibitory of TGFβ1 to Claudin 1 in A549 cells could be cell type specific. The basal expression level of Claudin 1 mRNA was increased in a time-dependent manner between 24 and 48 h, which was significantly enhanced by TNFα stimulation as determined by real-time quantitative RT-PCR (Figure 2D).

Furthermore, immunofluorescent staining demonstrated that TNFα-induced Claudin 1 protein expression was mainly found in the cytoplasm and not at the boundary of cell-to-cell contacts (Figure 3A). The cytosolic distribution of Claudin 1 was further demonstrated with confocal microscopy at higher magnification (Figure 3B). Assembly of tight junctions recruits tight junction proteins into complexes; therefore, make them resistant to detergent-salt extractions. Conversely, disassembly of tight junction may result in internalization or diffuse cytoplasmic distribution of tight junction proteins, making them more extractable with detergent salt solutions [34]. Thus, to further determine the distribution of Claudin 1, we used Triton-X100 extraction followed by western blotting. Claudin 1 was found mainly in Triton X-100 soluble cytoplasm fractions, and increased after 24 h of TNFα stimulation. In contrast, ZO-1 and Occludin (epithelial surface markers) were mainly found in Triton X-100 insoluble precipitates. GAPDH, a cytosolic protein was found mainly in Triton X-100 soluble fraction (Figure 3C). Therefore, our results suggest that the increased Claudin 1 is not mainly in tight junction but diffusely distributed in the cytoplasm.

### Knocking down Claudin 1 with siRNA significantly blocked TNFα-induced gene expression

To determine the role of Claudin 1 in TNFα related cellular functions, we used microarray to analyze gene expression profiles in cells treated with or without TNFα and in the presence of Claudin 1 siRNA or control siRNA. TNFα stimulation (20 ng/ml for 24 h) increased Claudin 1 mRNA expression, while Claudin 1 siRNA effectively reduced both basal level and TNFα-induced Claudin 1 gene expression (Figure 4A). Interestingly, TNFα reduced Claudin 2 gene expression, which was not affected by Claudin 1 siRNA treatment. TNFα has no significant effects on other Claudin family members (Table S1). Principle Component Analysis (PCA) indicated that the overall gene expression patterns were clearly separated based on either the TNFα stimulation or Claudin 1 siRNA pre-treatments (Figure 4B). Hierarchical cluster analysis showed that the down regulation of Claudin 1 with siRNA has a profound effect on the gene expression profile. A group of genes were up-regulated in Claudin 1 siRNA treated cells, and another group of genes were down-regulated, regardless of TNFα treatment (Figure 4C).

As expected, TNFα stimulation altered expression of many genes. One group of genes was up-regulated by TNFα in both Control and Claudin 1 siRNA transfected cells (Figure 4C, red arrow). However, another group of genes regulated by TNFα in Control siRNA transfected cells are less regulated in Claudin 1 siRNA transfected cells (Figure 4C, blue arrow). Significance Analysis of Microarray (SAM) was performed to detect genes significantly changed by TNFα treatment, which is indicated by False Discovery Rate (FDR) q value less than 5.0% and fold change greater than 1.3. In control siRNA transfected cells, TNFα changed expression of 468 genes, of which 450 genes were up-regulated, whereas only 18 genes were down-regulated. In contrast, in Claudin 1 siRNA transfected cells, only 123 genes were significantly changed by TNFα, and all of them were up-regulated. The Venn diagram revealed that 353 of the genes altered by TNFα in Control siRNA transfected cells are not changed in Claudin 1 siRNA transfected cells. This means that knock-down of Claudin 1 blocked 75% of the TNFα-induced gene expression changes (Figure 4D). The top 20 genes up-regulated by TNFα are listed in Table 1. The folds of changes were decreased in Claudin 1 siRNA treated group in most of these genes. The top 20 down-regulated genes by TNFα are listed in Table 2. None of them remains significantly changed in Claudin 1 siRNA treated group. Six genes from up- or down-regulated genes were verified with qRT-PCR. Similar trends between microarray and qRT-PCR were observed (Figure 5). These results indicate that Claudin 1 plays an important role in mediating TNFα-induced gene expression.

### Claudin 1 siRNA blocked TNFα-induced genes are related to cell migration

We further analyzed Bio-functions of the 353 genes, which were blocked by Claudin 1 siRNA (highlighted area with dots on Venn diagram in Figure 4D). Among these significantly changed genes, 103 genes showed Bio functions rerated to Cellular Development, Cellular Movement, Cell-To-Cell Signaling and Interaction, Tumor Morphology and/or Cell Morphology (Table S2). Ingenuity Pathway Analysis shows that the top ranked functional networks blocked by Claudin 1 siRNA treatment are related to antigen presentation, cellular development, gene expression, cellular movement and cell death (Table 3). In two of these functional networks, NFκB is well connected as a central hub of multiple molecules. One of them is related to antigen presentation (data now shown) and another is related to inflammation (Figure 6A, Table S3A). Recent studies have demonstrated that NFκB promotes EMT, migration and invasion in cancer cells [14], [43], [44]. In the functional network on Cellular Movement, MMP-9 is located in the center (Figure 6B, Table S3B). MMP-9 is one of the most important proteases for human lung epithelial cell migration [27], [45].

### Claudin 1 is important for TNFα-induced cell migration in A549 cells

To determine whether Claudin 1 is involved in TNFα-induced cell migration, we first compared the role of TNFα and TGFβ1 in cell migration with a wound-healing assay. TNFα plus TGFβ1 treatment significantly increased motility in A549 cells (Figure 7A and 7B). TNFα alone, but not TGFβ1, increased wound closure as effectively as TNFα plus TGFβ1 did (Figure 7B). To determine the increased wound closure is due to increase cell proliferation and/or migration, we examined the effects of TNFα and/or TGFβ on cell proliferation with MTS assay. Neither or both of them affected cell proliferation within 24 h of treatment (Figure 7C). Microarray and qRT-PCR demonstrated that TNFα increased MMP-9 gene expression. A gelatin zymography assay demonstrated that the level of active-MMP 9 was increased by TNFα treatment; while the levels of both pro- and active-MMP 2 had no dramatic changes (Figure 7D). These results indicate that TNFα is responsible for increased cell migration.

Transfection of cells with Claudin 1 siRNA effectively reduced both the basal and TNFα-induced expression of Claudin 1 mRNA (Table S1) and protein (Figure 8C) levels and cell migration (Figure 8A and 8B). Next, we transfected A549 cells with Claudin 1-pcDNA, which effectively enhanced cell migration (Figure 8D and 8E). Western blotting confirmed that Claudin 1-pcDNA transfection increased the expression level of Claudin 1 protein (Figure 8F). Collectively, these results indicate that Claudin 1 has important role in mediating TNFα-induced cell migration.

To determine whether the role of Claudin 1 in TNFα-induced cell migration is specific, cells were serum-starved for 3 h and then stimulated with 10% serum and/or EGF (50 ng/ml). Serum plus EGF significantly increased cell migration. Interestingly, Claudin 1 siRNA transfected cells showed significantly increased migration in either EGF or serum treated group. This is more significant in cells treated with both serum and EGF (Figure 9A). We then performed western blotting, serum and/or EGF slightly increased Claudin 1 protein levels. Claudin 1 siRNA effectively reduced the levels of Claudin 1 (Figure 9B). To test whether down-regulation of Claudin 1 can alter TNFα-induced cell morphology changes, cells were treated with control or Claudin 1 siRNA and stimulated with TNFα, and/or TGFβ.Claudin 1 siRNA reduced the morphological changes induced by TNFα alone or by TNFα plus TGFβ, and TGFβ alone had little effects on stress fiber formation and cell morphology, as shown by F-actin staining (Figure 9C).

## Discussion

One of the novel findings of the present study is the evidence that in human lung cancer A549 cells TNFα alone induced morphological changes, stress fiber formation, cell migration and the alteration of gene expression. More importantly, we found that these cellular functions are largely mediated through the induction of Claudin 1.

TNFα is known to augment TGFβ1-induced EMT in various cells [12], [13], [46], [47]. To investigate EMT in A549 cells, we treated cells with TNFα, TGFβ1 or in combination, and examined the morphological changes, cytoskeletal structure and expression of EMT markers. Through these experiments, we found that TNFα and TGFβ1 may have different roles in these human lung cancer cells. TGFβ1 is more effectively inhibiting expression of E-Cadherin, a marker for the differentiation of epithelial cells, whereas TNFα is more effective in increasing Claudin 1 expression and through Claudin 1 to mediate down-stream gene expression and cell migration. It has been shown that TNFα stimulated EMT of human colonic organoids [11], promoted EMT in renal carcinoma cells [48], [49] and in human skin cells [50]. In A549 cells, TNFα alone induced cell morphological changes and cell migration. Although these changes are related to EMT, we do not have enough evidence to support TNFα alone induced EMT in this cell type.

Normally, Claudin 1 expresses in lung epithelial cells and regulates tight junction permeability [51]. Claudin 1 expression is generally known to be decreased by TNFα, and the decreased protein expression and protein redistribution of Claudin 1 lead to the decrease in the trans-epithelial electric resistance and the increase in the paracellular permeability of epithelial cells [52], [53]. TNFα increased Claudin 1 expression in human pancreatic cancer cells [54], and in airway smooth muscle cells [55]. In the present study, TNFα strongly increased Claudin 1 expression in human lung cancer cells. The increased Claudin 1 is mainly in Triton-soluble cytoplasm, not in the tight junction complex of Triton-insoluble fraction. More importantly, knock-down of Claudin 1 blocked 75% of the TNFα-induced gene expression changes. Two of the top five TNFα-induced functional networks effectively blocked by Claudin 1 siRNA are related to NFκB. It has been shown that NFκB signaling is involved in EMT [14], [43], [44]. In the Table 1, we can see several cytokines and chemokines on the top of the gene list up-regulated by TNFα, such as CCL5, CCL2, IL-8, IL-6 and IL-1α. The folds of changes of these genes were significantly lower in Claudin 1 siRNA treated cells. In the NFκB related inflammatory signal network (Figure 6A, Table S3A), genes encoding proteins related to inflammation can be identified, such as CXCL10 (chemokine CXC motif ligand 10), CSF2 (colony stimulating factor 2), PLA2G4A and PLA2G4C (phospholipase A2, group IV members), KLRK1 and KLRC3 (killer cell lectin-like receptor subfamily members), and S100A3 (S100 calcium binding protein A3). TNFα is one of the most important inflammatory mediators in tumorigenesis. Our results suggest that Claudin 1 may be a crucial mediator in TNFα-initiated inflammatory responses.

In the signal network related to cell migration, genes are centered on MMP-9 (Figure 6B, Table S3B). Indeed, the gene expression, protein level and activity of MMP-9 were increased after TNFα stimulation. PLAT, PLAU and PLAUR (stands for plasminogen activator, tissue type, urokinase type and PLAU receptor, respectively) are among the genes blocked by Claudin 1 siRNA. They are important players in the cell migration. Collagen type IV alpha-1 (COL4A1) and nidogen-2 (NID2) are components of the basement membrane, and may play a role in cell interactions with extracellular matrix. Furthermore, over 100 genes listed in Table S2 are related to cellular movements, cell-to-cell interaction, tumor morphology and cell morphology. These data indicate the importance of Claudin 1 in mediating TNFα related lung cancer cell migration. The increased expression of Claudin-1 in colon cancer cells resulted in increased tumor growth and metastasis in vivo, whereas the siRNA knock-down of Claudin 1 in metastatic colon cancer cells inhibited migration and invasion [23]. Similarly, Claudin 1 over-expression increased cell motility in oral squamous cell carcinoma, melanoma and hepatocellular carcinoma [24]–[26].

In contrast to these findings, in human lung cancer CL1–5 cells, over expression of Claudin 1 inhibited cell migration, whereas knockdown of Claudin 1 restored the migration and invasive ability of cells with stably transfection of Claudin 1 [56]. In the present study, Claudin 1 siRNA transfection enhanced cell migration stimulated by EGF and/or serum in A549 cells (Figure 9). As a TJ protein Claudin 1 participates in the cell-to-cell adhesion. Its down-regulation may reduce the TJs and thus, promote cell migration. TNFα-induced Claudin 1, on other hand is mainly in the cytoplasm, and is involved in TNFα-induced gene expression. Since many of these Claudin 1 dependent genes are related to cell movement and morphology, Claudin 1 may mediate TNFα-initiated cell migration with multiple mechanisms.

In summary, we found that TNFα stimulation induces the gene expression of Claudin 1 in human lung cancer cells, and the latter acts as the signal mediator to regulate gene expression and cell migration. Further study on this pathway may serve as a mean to develop a novel therapeutic target for cancer.

## Supporting Information

Table S1 (DOCX)

Table S2 (DOCX)

Table S3 (DOC)

## References

[1] Mantovani A, Allavena P, Sica A, Balkwill F (2008). Cancer-related inflammation.. Nature.

[2] Balkwill F, Charles KA, Mantovani A (2005). Smoldering and polarized inflammation in the initiation and promotion of malignant disease.. Cancer Cell.

[3] Balkwill F (2009). Tumour necrosis factor and cancer.. Nat Rev Cancer.

[4] Malik ST, Griffin DB, Fiers W, Balkwill FR (1989). Paradoxical effects of tumour necrosis factor in experimental ovarian cancer.. Int J Cancer.

[5] Malik ST, Naylor MS, East N, Oliff A, Balkwill FR (1990). Cells secreting tumour necrosis factor show enhanced metastasis in nude mice.. Eur J Cancer.

[6] Arnott CH, Scott KA, Moore RJ, Robinson SC, Thompson RG (2004). Expression of both TNF-alpha receptor subtypes is essential for optimal skin tumour development.. Oncogene.

[7] Knight B, Yeoh GC, Husk KL, Ly T, Abraham LJ (2000). Impaired preneoplastic changes and liver tumor formation in tumor necrosis factor receptor type 1 knockout mice.. J Exp Med.

[8] Kitakata H, Nemoto-Sasaki Y, Takahashi Y, Kondo T, Mai M (2002). Essential roles of tumor necrosis factor receptor p55 in liver metastasis of intrasplenic administration of colon 26 cells.. Cancer Res.

[9] Balkwill F (2002). Tumor necrosis factor or tumor promoting factor?. Cytokine Growth Factor Rev.

[10] Harrison ML, Obermueller E, Maisey NR, Hoare S, Edmonds K (2007). Tumor necrosis factor alpha as a new target for renal cell carcinoma: two sequential phase II trials of infliximab at standard and high dose.. J Clin Oncol.

[11] Bates RC, Mercurio AM (2003). Tumor necrosis factor-alpha stimulates the epithelial-to-mesenchymal transition of human colonic organoids.. Mol Biol Cell.

[12] Takahashi E, Nagano O, Ishimoto T, Yae T, Suzuki Y (2010). Tumor necrosis factor-alpha regulates transforming growth factor-beta-dependent epithelial-mesenchymal transition by promoting hyaluronan-CD44-moesin interaction.. J Biol Chem.

[13] Yamauchi Y, Kohyama T, Takizawa H, Kamitani S, Desaki M (2010). Tumor necrosis factor-alpha enhances both epithelial-mesenchymal transition and cell contraction induced in A549 human alveolar epithelial cells by transforming growth factor-beta1.. Exp Lung Res.

[14] Chua HL, Bhat-Nakshatri P, Clare SE, Morimiya A, Badve S (2007). NF-kappaB represses E-cadherin expression and enhances epithelial to mesenchymal transition of mammary epithelial cells: potential involvement of ZEB-1 and ZEB-2.. Oncogene.

[15] Medici D, Hay ED, Goodenough DA (2006). Cooperation between snail and LEF-1 transcription factors is essential for TGF-beta1-induced epithelial-mesenchymal transition.. Mol Biol Cell.

[16] Ikenouchi J, Matsuda M, Furuse M, Tsukita S (2003). Regulation of tight junctions during the epithelium-mesenchyme transition: direct repression of the gene expression of claudins/occludin by Snail.. J Cell Sci.

[17] Ohkubo T, Ozawa M (2004). The transcription factor Snail downregulates the tight junction components independently of E-cadherin downregulation.. J Cell Sci.

[18] Bruewer M, Luegering A, Kucharzik T, Parkos CA, Madara JL (2003). Proinflammatory cytokines disrupt epithelial barrier function by apoptosis-independent mechanisms.. J Immunol.

[19] Coyne CB, Vanhook MK, Gambling TM, Carson JL, Boucher RC (2002). Regulation of airway tight junctions by proinflammatory cytokines.. Mol Biol Cell.

[20] Hewitt KJ, Agarwal R, Morin PJ (2006). The claudin gene family: expression in normal and neoplastic tissues.. BMC Cancer.

[21] Kominsky SL (2006). Claudins: emerging targets for cancer therapy.. Expert Rev Mol Med.

[22] Swisshelm K, Macek R, Kubbies M (2005). Role of claudins in tumorigenesis.. Adv Drug Deliv Rev.

[23] Dhawan P, Singh AB, Deane NG, No Y, Shiou SR (2005). Claudin-1 regulates cellular transformation and metastatic behavior in colon cancer.. J Clin Invest.

[24] Leotlela PD, Wade MS, Duray PH, Rhode MJ, Brown HF (2007). Claudin-1 overexpression in melanoma is regulated by PKC and contributes to melanoma cell motility.. Oncogene.

[25] dos Reis PP, Bharadwaj RR, Machado J, Macmillan C, Pintilie M (2008). Claudin 1 overexpression increases invasion and is associated with aggressive histological features in oral squamous cell carcinoma.. Cancer.

[26] Yoon CH, Kim MJ, Park MJ, Park IC, Hwang SG (2010). Claudin-1 acts through c-Abl-protein kinase Cdelta (PKCdelta) signaling and has a causal role in the acquisition of invasive capacity in human liver cells.. J Biol Chem.

[27] Xiao H, Bai X, Lu W, Kapus A, Mak AS (2010). PKC cascade regulates recruitment of MMP-9 to podosomes and its release and activation.. Mol Cell Biol.

[28] Lodyga M, Bai XH, Kapus A, Liu M (2010). Adaptor protein XB130 is a Rac-controlled component of lamellipodia that regulates cell motility and invasion.. J Cell Sci.

[29] Xiao H, Eves R, Yeh C, Kan W, Xu F (2009). Phorbol ester-induced podosomes in normal human bronchial epithelial cells.. J Cell Physiol.

[30] Han B, Bai XH, Lodyga M, Xu J, Yang BB (2004). Conversion of mechanical force into biochemical signaling.. J Biol Chem.

[31] Han B, Mura M, Andrade CF, Okutani D, Lodyga M (2005). TNFalpha-induced long pentraxin PTX3 expression in human lung epithelial cells via JNK.. J Immunol.

[32] Xu J, Bai XH, Lodyga M, Han B, Xiao H (2007). XB130, a novel adaptor protein for signal transduction.. J Biol Chem.

[33] Lodyga M, De Falco V, Bai XH, Kapus A, Melillo RM (2009). XB130, a tissue-specific adaptor protein that couples the RET/PTC oncogenic kinase to PI 3-kinase pathway.. Oncogene.

[34] Nishiyama R, Sakaguchi T, Kinugasa T, Gu X, MacDermott RP (2001). Interleukin-2 receptor beta subunit-dependent and -independent regulation of intestinal epithelial tight junctions.. J Biol Chem.

[35] Tokes AM, Kulka J, Paku S, Szik A, Paska C (2005). Claudin-1, -3 and -4 proteins and mRNA expression in benign and malignant breast lesions: a research study.. Breast Cancer Res.

[36] Shiozaki A, Lodyga M, Bai X, Nadesalingam J, Oyaizu T (2011). XB130, a novel adaptor protein, promotes thyroid tumor growth.. Am J Pathol.

[37] dos Santos CC, Han B, Andrade CF, Bai X, Uhlig S (2004). DNA microarray analysis of gene expression in alveolar epithelial cells in response to TNFalpha, LPS, and cyclic stretch.. Physiol Genomics.

[38] dos Santos CC, Okutani D, Hu P, Han B, Crimi E (2008). Differential gene profiling in acute lung injury identifies injury-specific gene expression.. Crit Care Med.

[39] Mura M, Han B, Andrade CF, Seth R, Hwang D (2006). The early responses of VEGF and its receptors during acute lung injury: implication of VEGF in alveolar epithelial cell survival.. Crit Care.

[40] Tusher VG, Tibshirani R, Chu G (2001). Significance analysis of microarrays applied to the ionizing radiation response.. Proc Natl Acad Sci U S A.

[41] Kim KB, Yi JS, Nguyen N, Lee JH, Kwon YC (2011). Cell-surface receptor for complement component C1q (gC1qR) is a key regulator for lamellipodia formation and cancer metastasis.. J Biol Chem.

[42] Tang PS, Tsang ME, Lodyga M, Bai XH, Miller A (2006). Lipopolysaccharide accelerates caspase-independent but cathepsin B-dependent death of human lung epithelial cells.. J Cell Physiol.

[43] Maier HJ, Schmidt-Strassburger U, Huber MA, Wiedemann EM, Beug H (2010). NF-kappaB promotes epithelial-mesenchymal transition, migration and invasion of pancreatic carcinoma cells.. Cancer Lett.

[44] Dong R, Wang Q, He XL, Chu YK, Lu JG (2007). Role of nuclear factor kappa B and reactive oxygen species in the tumor necrosis factor-alpha-induced epithelial-mesenchymal transition of MCF-7 cells.. Braz J Med Biol Res.

[45] Legrand C, Gilles C, Zahm JM, Polette M, Buisson AC, Kaplan H (1999). Airway epithelial cell migration dynamics. MMP-9 role in cell-extracellular matrix remodeling.. J Cell Biol.

[46] Liu X (2008). Inflammatory cytokines augments TGF-beta1-induced epithelial-mesenchymal transition in A549 cells by up-regulating TbetaR-I.. Cell Motil Cytoskeleton.

[47] Willis BC, Liebler JM, Luby-Phelps K, Nicholson AG, Crandall ED (2005). Induction of epithelial-mesenchymal transition in alveolar epithelial cells by transforming growth factor-beta1: potential role in idiopathic pulmonary fibrosis.. Am J Pathol.

[48] Chuang MJ, Sun KH, Tang SJ, Deng MW, Wu YH (2008). Tumor-derived tumor necrosis factor-alpha promotes progression and epithelial-mesenchymal transition in renal cell carcinoma cells.. Cancer Sci.

[49] Wu ST, Sun GH, Hsu CY, Huang CS, Wu YH (2011). Tumor necrosis factor-alpha induces epithelial-mesenchymal transition of renal cell carcinoma cells via a nuclear factor kappa B-independent mechanism.. Exp Biol Med (Maywood).

[50] Yan C, Grimm WA, Garner WL, Qin L, Travis T (2010). Epithelial to mesenchymal transition in human skin wound healing is induced by tumor necrosis factor-alpha through bone morphogenic protein-2.. Am J Pathol.

[51] Coyne CB, Gambling TM, Boucher RC, Carson JL, Johnson LG (2003). Role of claudin interactions in airway tight junctional permeability.. Am J Physiol Lung Cell Mol Physiol.

[52] Baker OJ, Camden JM, Redman RS, Jones JE, Seye CI (2008). Proinflammatory cytokines tumor necrosis factor-alpha and interferon-gamma alter tight junction structure and function in the rat parotid gland Par-C10 cell line.. Am J Physiol Cell Physiol.

[53] Poritz LS, Garver KI, Tilberg AF, Koltun WA (2004). Tumor necrosis factor alpha disrupts tight junction assembly.. J Surg Res.

[54] Kondo J, Sato F, Kusumi T, Liu Y, Motonari O (2008). Claudin-1 expression is induced by tumor necrosis factor-alpha in human pancreatic cancer cells.. Int J Mol Med.

[55] Fujita H, Chalubinski M, Rhyner C, Indermitte P, Meyer N (2011). Claudin-1 expression in airway smooth muscle exacerbates airway remodeling in asthmatic subjects.. J Allergy Clin Immunol 127: 1612–1621.

[56] Chao YC, Pan SH, Yang SC, Yu SL, Che TF (2009). Claudin-1 is a metastasis suppressor and correlates with clinical outcome in lung adenocarcinoma.. Am J Respir Crit Care Med.

