# Supplementary material for: Claudin 1 Mediates TNFα-Induced Gene Expression and Cell Migration in Human Lung Carcinoma Cells
Source: PLoS One. 2012 May 31;7(5):e38049. doi: 10.1371/journal.pone.0038049 (PMC3365005; doi:10.1371/journal.pone.0038049)
Supplement: Table S1 — (DOCX) [file pone.0038049.s001.docx]

**Table S1. Effects of TNFα on gene expression of Claudin family members in Control-siRNA transfected A549 cells**

| Gene Symbol | Gene ID | Fold Change | q-value (%) |
| --- | --- | --- | --- |
| CLDN1 | NM_021101 | 1.606 | <0.001 |
| CLDN2 | NM_020384 | -1.543 | 3.186 |
| CLDN3 | NM_001306 | 1.084 | ≥5.0 |
| CLDN4 | NM_001305 | -1.016 | ≥5.0 |
| CLDN5 | NM_003277 | -1.007 | ≥5.0 |
| CLDN6 | NM_021195 | -1.001 | ≥5.0 |
| CLDN7 | NM_001307 | -1.057 | ≥5.0 |
| CLDN8 | NM_199328 | 1.103 | ≥5.0 |
| CLDN9 | NM_020982 | -1.010 | ≥5.0 |
| CLDN10 | NM_182848 | -1.167 | ≥5.0 |
| CLDN11 | NM_005602 | -1.080 | ≥5.0 |
| CLDN12 | NM_012129 | 1.224 | ≥5.0 |
| CLDN14 | NM_144492 | -1.013 | ≥5.0 |
| CLDN15 | NM_014343 | -1.019 | ≥5.0 |
| CLDN16 | NM_006580 | 1.214 | ≥5.0 |
| CLDN17 | NM_012131 | -1.003 | ≥5.0 |
| CLDN18 | NM_001002026 | -1.083 | ≥5.0 |
| CLDN19 | NM_001123395 | -1.044 | ≥5.0 |
| CLDN20 | NM_001001346 | 1.080 | ≥5.0 |
| CLDN22 | NM_001111319 | -1.041 | ≥5.0 |
| CLDN23 | NM_194284 | 1.015 | ≥5.0 |
