# Supplementary material for: Claudin 1 Mediates TNFα-Induced Gene Expression and Cell Migration in Human Lung Carcinoma Cells
Source: PLoS One. 2012 May 31;7(5):e38049. doi: 10.1371/journal.pone.0038049 (PMC3365005; doi:10.1371/journal.pone.0038049)
Supplement: Table S2 — (DOCX) [file pone.0038049.s002.docx]

**Table S2. Specific Bio functions (Cellular Development, Cellular Movement, Cell-To-Cell Signaling and Interaction, Tumor Morphology or Cell Morphology-related) of significantly changed genes by TNFα treatment in Control siRNA transfected cells, but not in Claudin 1 siRNA transfected cells**

| Up-Regulated Genes | | | | | | | | | |
| --- | --- | --- | --- | --- | --- | --- | --- | --- | --- |
| Gene Symbol | Gene Name | Gene ID | Bio functions | | | | | Fold Change | q-value (%) |
|  |  |  | Cellular Development | Cellular Movement | Cell-To-Cell Signaling and Interaction | Tumor Morphology | Cell Morphology |  |  |
| EFNA1 | ephrin-A1 | NM_004428 | ● | ● | ● | ● | ● | 3.819 | <0.001 |
| IL32 | interleukin 32 | NM_001012631 |  | ● | ● |  |  | 2.982 | <0.001 |
| IFIH1 | interferon induced with helicase C domain 1 | NM_022168 | ● |  |  |  |  | 2.423 | <0.001 |
| CXCL10 | chemokine (C-X-C motif) ligand 10 | NM_001565 | ● | ● | ● |  |  | 2.350 | <0.001 |
| BDKRB1 | bradykinin receptor B1 | NM_000710 |  | ● | ● |  |  | 2.188 | <0.001 |
| HMGA2 | high mobility group AT-hook 2 | NM_003483 | ● |  |  |  |  | 2.082 | <0.001 |
| PTGS2 | prostaglandin-endoperoxide synthase 2 (prostaglandin G/H synthase and cyclooxygenase) | NM_000963 | ● | ● | ● | ● |  | 2.062 | <0.001 |
| CD274 | CD274 molecule | NM_014143 |  |  | ● | ● |  | 1.953 | 0.387 |
| MSC | musculin (activated B-cell factor-1) | NM_005098 | ● |  |  |  |  | 1.894 | <0.001 |
| TLR3 | toll-like receptor 3 | NM_003265 | ● | ● | ● |  |  | 1.816 | <0.001 |
| PCDHB6 | protocadherin beta 6 | NM_018939 |  |  | ● |  |  | 1.797 | 0.387 |
| BDKRB2 | bradykinin receptor B2 | NM_000623 | ● |  |  | ● | ● | 1.787 | <0.001 |
| MMP9 | matrix metallopeptidase 9 (gelatinase B, 92kDa gelatinase, 92kDa type IV collagenase) | NM_004994 | ● | ● | ● | ● |  | 1.785 | 0.387 |
| IRF1 | interferon regulatory factor 1 | NM_002198 | ● |  |  | ● |  | 1.783 | <0.001 |
| HLA-G | major histocompatibility complex, class I, G | NM_002127 |  | ● | ● |  |  | 1.765 | <0.001 |
| MR1 | major histocompatibility complex, class I-related | NM_001531 | ● |  |  |  |  | 1.764 | <0.001 |
| PPAP2B | phosphatidic acid phosphatase type 2B | NM_003713 |  | ● | ● |  |  | 1.760 | <0.001 |
| KLRC3 | killer cell lectin-like receptor subfamily C, member 3 | NM_002261 |  |  | ● |  |  | 1.758 | 0.595 |
| OPTN | optineurin | NM_001008211 | ● |  |  |  |  | 1.737 | <0.001 |
| RELB | v-rel reticuloendotheliosis viral oncogene homolog B | NM_006509 | ● |  | ● |  |  | 1.732 | <0.001 |
| HLA-A | major histocompatibility complex, class I, A | NM_002116 |  |  | ● |  |  | 1.710 | <0.001 |
| RND1 | Rho family GTPase 1 | NM_014470 |  |  | ● |  | ● | 1.709 | 0.387 |
| STAT5A | signal transducer and activator of transcription 5A | NM_003152 | ● | ● | ● |  |  | 1.705 | 0.387 |
| RARRES1 | retinoic acid receptor responder (tazarotene induced) 1, transcript variant 1 | NM_206963 |  | ● |  |  |  | 1.705 | 0.387 |
| MTSS1 | metastasis suppressor 1 | NM_014751 |  | ● |  |  |  | 1.688 | 0.387 |
| CA2 | carbonic anhydrase II | NM_000067 | ● |  |  |  |  | 1.686 | 0.857 |
| VDR | vitamin D (1,25- dihydroxyvitamin D3) receptor | NM_001017535 | ● | ● |  |  |  | 1.660 | 0.387 |
| CSF2 | colony stimulating factor 2 (granulocyte-macrophage) | NM_000758 | ● | ● | ● | ● | ● | 1.648 | 3.152 |
| NID2 | nidogen 2 (osteonidogen) | NM_007361 | ● |  | ● |  |  | 1.623 | <0.001 |
| XDH | xanthine dehydrogenase | NM_000379 | ● | ● | ● |  | ● | 1.616 | 0.387 |
| PARP9 | poly (ADP-ribose) polymerase family, member 9 | NM_031458 |  | ● |  |  |  | 1.615 | <0.001 |
| CLDN1 | claudin 1 | NM_021101 |  |  | ● |  |  | 1.605 | <0.001 |
| PTPRE | protein tyrosine phosphatase, receptor type, E | NM_006504 |  |  |  | ● |  | 1.584 | 0.595 |
| IFNGR1 | interferon gamma receptor 1 | NM_000416 |  |  | ● |  |  | 1.571 | 0.387 |
| PSMB9 | proteasome (prosome, macropain) subunit, beta type, 9 (large multifunctional peptidase 2) | NM_002800 |  |  | ● |  |  | 1.564 | <0.001 |
| TAPBP | TAP binding protein (tapasin) | NM_172208 | ● |  |  |  |  | 1.554 | <0.001 |
| RARRES3 | retinoic acid receptor responder (tazarotene induced) 3 | NM_004585 | ● |  | ● |  |  | 1.535 | 0.595 |
| PSME2 | proteasome (prosome, macropain) activator subunit 2 (PA28 beta) | NM_002818 |  |  | ● |  |  | 1.527 | <0.001 |
| ROBO1 | roundabout, axon guidance receptor, homolog 1 (Drosophila), transcript variant 2 | NM_133631 | ● | ● | ● |  | ● | 1.524 | <0.001 |
| RASGRP1 | RAS guanyl releasing protein 1 (calcium and DAG-regulated), transcript variant 1 | NM_005739 | ● | ● | ● |  |  | 1.523 | <0.001 |
| SRGN | serglycin | NM_002727 | ● |  |  |  | ● | 1.521 | <0.001 |
| PSMB8 | proteasome (prosome, macropain) subunit, beta type, 8 (large multifunctional peptidase 7), transcript variant 1 | NM_004159 |  |  | ● |  |  | 1.512 | 0.595 |
| EBF1 | early B-cell factor 1 | NM_024007 | ● | ● |  |  |  | 1.510 | 0.595 |
| CHST2 | carbohydrate (N-acetylglucosamine-6-O) sulfotransferase 2 | NM_004267 |  | ● |  |  |  | 1.502 | 0.387 |
| RASGRF2 | Ras protein-specific guanine nucleotide-releasing factor 2 | NM_006909 | ● |  | ● |  |  | 1.493 | 0.595 |
| VNN1 | vanin 1 | NM_004666 |  | ● | ● |  |  | 1.493 | 0.857 |
| TFF1 | trefoil factor 1 | NM_003225 | ● | ● |  | ● |  | 1.493 | 2.306 |
| ECE1 | endothelin converting enzyme 1 | NM_001397 | ● |  |  |  |  | 1.477 | 0.595 |
| PLAUR | plasminogen activator, urokinase receptor | NM_002659 | ● | ● | ● | ● | ● | 1.476 | 0.595 |
| ANPEP | alanyl (membrane) aminopeptidase | NM_001150 | ● | ● |  |  |  | 1.475 | 0.387 |
| TMOD1 | tropomodulin 1 | NM_003275 |  |  | ● |  |  | 1.471 | 0.857 |
| IL7 | interleukin 7 | NM_000880 | ● | ● | ● | ● | ● | 1.471 | 0.595 |
| ABCA1 | ATP-binding cassette, sub-family A (ABC1), member 1 | NM_005502 |  |  | ● |  |  | 1.471 | <0.001 |
| ADAM19 | ADAM metallopeptidase domain 19 (meltrin beta) | NM_033274 |  | ● |  |  |  | 1.470 | 0.387 |
| CXCL6 | chemokine (C-X-C motif) ligand 6 (granulocyte chemotactic protein 2) | NM_002993 |  | ● | ● | ● |  | 1.468 | 1.627 |
| PODXL | podocalyxin-like | NM_001018111 |  | ● | ● |  |  | 1.467 | 1.627 |
| RAB27A | RAB27A, member RAS oncogene family | NM_004580 | ● |  |  |  |  | 1.460 | <0.001 |
| PLAU | plasminogen activator, urokinase | NM_002658 | ● | ● | ● | ● | ● | 1.460 | 0.387 |
| IL23A | interleukin 23, alpha subunit p19 | NM_016584 | ● |  | ● |  |  | 1.453 | 0.595 |
| PLAT | plasminogen activator, tissue | NM_000930 |  | ● | ● | ● |  | 1.451 | 1.627 |
| NAB1 | NGFI-A binding protein 1 (EGR1 binding protein 1) | NM_005966 | ● |  |  |  |  | 1.449 | 2.306 |
| DFNA5 | deafness, autosomal dominant 5 | NM_004403 | ● |  |  |  |  | 1.448 | 0.595 |
| IFI16 | interferon, gamma-inducible protein 16 | NM_005531 | ● |  |  |  | ● | 1.442 | 3.186 |
| CXCL11 | chemokine (C-X-C motif) ligand 11 | NM_005409 | ● | ● | ● |  |  | 1.440 | 2.306 |
| TNC | tumor necrosis factor, alpha-induced protein 2 | NM_006291 | ● | ● | ● |  |  | 1.440 | 0.387 |
| PLA2G4A | phospholipase A2, group IVA (cytosolic, calcium-dependent) | NM_024420 |  | ● |  |  | ● | 1.439 | 0.595 |
| HIVEP2 | human immunodeficiency virus type I enhancer binding protein 2 | NM_006734 | ● |  |  |  |  | 1.437 | 3.818 |
| IL4R | interleukin 4 receptor | NM_000418 | ● | ● | ● |  |  | 1.437 | <0.001 |
| KLRK1 | killer cell lectin-like receptor subfamily K, member 1 | NM_007360 |  |  | ● |  |  | 1.436 | 2.075 |
| IFNAR2 | interferon (alpha, beta and omega) receptor 2 | NM_207585 |  |  | ● |  |  | 1.433 | 0.387 |
| NFKBIZ | nuclear factor of kappa light polypeptide gene enhancer in B-cells inhibitor, zeta, transcript variant 1 | NM_031419 |  | ● |  |  |  | 1.432 | 0.387 |
| COL4A1 | collagen, type IV, alpha 1 | NM_001845 | ● | ● |  | ● |  | 1.430 | 0.387 |
| VEGFC | vascular endothelial growth factor C | NM_005429 | ● | ● | ● | ● | ● | 1.426 | 0.387 |
| CEBPB | CCAAT/enhancer binding protein (C/EBP), beta | NM_005194 | ● |  | ● | ● |  | 1.422 | 0.595 |
| BID | BH3 interacting domain death agonist | NM_197966 |  |  |  |  | ● | 1.422 | 1.060 |
| CSF2RA | colony stimulating factor 2 receptor, alpha, low-affinity (granulocyte-macrophage), transcript variant 1 | NM_006140 | ● | ● | ● | ● |  | 1.421 | 0.857 |
| C3AR1 | complement component 3a receptor 1 | NM_004054 |  |  | ● |  |  | 1.408 | 2.306 |
| TFPI2 | tissue factor pathway inhibitor 2 | NM_006528 |  | ● | ● |  |  | 1.404 | 0.857 |
| IFNGR2 | interferon gamma receptor 2 | NM_005534 | ● |  |  |  |  | 1.398 | 0.387 |
| ETS1 | ETS1 // v-ets erythroblastosis virus E26 oncogene homolog 1 (avian) | NM_005238 | ● | ● | ● |  |  | 1.388 | 2.075 |
| JUNB | jun B proto-oncogene | NM_002229 | ● | ● |  |  |  | 1.380 | 2.075 |
| B2M | beta-2-microglobulin | NM_004048 | ● |  | ● |  | ● | 1.380 | 0.387 |
| TPBG | trophoblast glycoprotein | NM_006670 |  | ● |  |  |  | 1.376 | 2.306 |
| IER3 | immediate early response 3 | NM_003897 |  |  |  | ● |  | 1.371 | 2.075 |
| NRP2 | neuropilin 2 | NM_201266 | ● | ● |  |  | ● | 1.371 | 0.595 |
| B4GALNT2 | beta-1,4-N-acetyl-galactosaminyl transferase 2 | NM_153446 |  |  | ● |  |  | 1.370 | 3.186 |
| SDC4 | syndecan 4 | NM_002999 | ● | ● | ● |  | ● | 1.370 | 1.060 |
| TAP1 | transporter 1, ATP-binding cassette, sub-family B (MDR/TAP) | NM_000593 |  |  |  |  | ● | 1.369 | 0.595 |
| PSME1 | proteasome (prosome, macropain) activator subunit 1 (PA28 alpha), transcript variant 2 | NM_176783 |  |  | ● |  |  | 1.362 | 1.060 |
| NFKB1 | nuclear factor of kappa light polypeptide gene enhancer in B-cells 1, transcript variant 1 | NM_003998 | ● | ● | ● |  | ● | 1.357 | 0.595 |
| PTPRO | protein tyrosine phosphatase, receptor type, O | NM_030667 |  | ● |  |  |  | 1.341 | 3.152 |
| MREG | melanoregulin | NM_018000 | ● |  |  |  |  | 1.338 | 2.306 |
| TDRD6 | tudor domain containing 6 | NM_001010870 | ● |  |  |  |  | 1.333 | 3.152 |
| CD47 | CD47 molecule | NM_001777 | ● | ● | ● | ● | ● | 1.329 | 2.075 |
| CDH2 | cadherin 2, type 1, N-cadherin (neuronal) | NM_001792 |  | ● | ● | ● |  | 1.329 | 3.152 |
| NCK1 | NCK adaptor protein 1 | NM_006153 | ● | ● | ● |  |  | 1.323 | 1.627 |
| IL18R1 | interleukin 18 receptor 1 | NM_003855 | ● |  | ● |  |  | 1.320 | 2.075 |
| NT5E | 5'-nucleotidase, ecto (CD73) | NM_002526 |  | ● |  |  |  | 1.316 | 1.060 |
| CIB1 | calcium and integrin binding 1 (calmyrin) | NM_006384 | ● | ● | ● |  |  | 1.312 | 2.306 |
| EREG | epiregulin | NM_001432 | ● | ● |  |  |  | 1.312 | 3.186 |
| CFLAR | CASP8 and FADD-like apoptosis regulator | NM_003879 |  |  |  |  | ● | 1.311 | 2.075 |
| FNDC3B | fibronectin type III domain containing 3B | NM_022763 | ● |  |  |  |  | 1.306 | 2.306 |
| B4GALT1 | UDP-Gal:betaGlcNAc beta 1,4- galactosyltransferase, polypeptide 1 | NM_001497 | ● | ● | ● |  |  | 1.303 | 1.627 |
