# Supplementary material for: Claudin 1 Mediates TNFα-Induced Gene Expression and Cell Migration in Human Lung Carcinoma Cells
Source: PLoS One. 2012 May 31;7(5):e38049. doi: 10.1371/journal.pone.0038049 (PMC3365005; doi:10.1371/journal.pone.0038049)
Supplement: Table S3 — (DOC) [file pone.0038049.s003.doc]

**Table S3A. Gene expression changes in Signal network related to inflammation (Figure 6A).**

|  | | | | |
| --- | --- | --- | --- | --- |
| Gene Symbol | Gene Name | Gene ID | Fold Change | q-value (%) |
| CXCL10 | chemokine (C-X-C motif) ligand 10 | NM_001565 | 2.350 | <0.001 |
| HMGA2 | high mobility group AT-hook 2 | NM_003483 | 2.082 | <0.001 |
| GCH1 | GTP cyclohydrolase 1 (dopa-responsive dystonia) | NM_000161 | 1.787 | 0.387 |
| KLRC3 | killer cell lectin-like receptor subfamily C, member 3 | NM_002261 | 1.758 | 0.595 |
| RELB | v-rel reticuloendotheliosis viral oncogene homolog B | NM_006509 | 1.732 | <0.001 |
| MTSS1 | metastasis suppressor 1 | NM_014751 | 1.688 | 0.387 |
| VDR | vitamin D (1,25- dihydroxyvitamin D3) receptor | NM_001017535 | 1.660 | 0.387 |
| CSF2 | colony stimulating factor 2 (granulocyte-macrophage) | NM_000758 | 1.648 | 3.152 |
| CYP3A4 | cytochrome P450, family 3, subfamily A, polypeptide 4 | NM_017460 | 1.584 | 2.075 |
| S100A3 | S100 calcium binding protein A3 | NM_002960 | 1.531 | 0.387 |
| PLA2G4C | phospholipase A2, group IVC (cytosolic, calcium-independent), transcript variant 1 | NM_003706 | 1.508 | 2.075 |
| TNC | tenascin C (hexabrachion) | NM_002160 | 1.496 | 2.306 |
| ABCA1 | ATP-binding cassette, sub-family A (ABC1), member 1 | NM_005502 | 1.471 | <0.001 |
| PLA2G4A | phospholipase A2, group IVA (cytosolic, calcium-dependent) | NM_024420 | 1.439 | 0.595 |
| KLRK1 | killer cell lectin-like receptor subfamily K, member 1 | NM_007360 | 1.436 | 2.075 |
| CEBPB | CCAAT/enhancer binding protein (C/EBP), beta | NM_005194 | 1.422 | 0.595 |
| TUBB2B | tubulin, beta 2B | NM_178012 | 1.411 | 3.186 |
| IER3 | immediate early response 3 | NM_003897 | 1.371 | 2.075 |
| P4HA3 | prolyl 4-hydroxylase, alpha polypeptide III | NM_182904 | 1.369 | 2.075 |
| NFKB1 | nuclear factor of kappa light polypeptide gene enhancer in B-cells 1, transcript variant 1 | NM_003998 | 1.357 | 0.595 |
| NT5E | 5'-nucleotidase, ecto (CD73) | NM_002526 | 1.316 | 1.060 |

**Table S3B. Gene expression changes in Signal network related to cell movement (Figure 6B).**

|  | | | | |
| --- | --- | --- | --- | --- |
| Gene Symbol | Gene Name | Gene ID | Fold Change | q-value (%) |
| MSC | musculin (activated B-cell factor-1) | NM_005098 | 1.894 | <0.001 |
| STEAP1 | six transmembrane epithelial antigen of the prostate 1 | NM_012449 | 1.881 | <0.001 |
| MMP9 | matrix metallopeptidase 9 (gelatinase B, 92kDa gelatinase, 92kDa type IV collagenase) | NM_004994 | 1.785 | 0.387 |
| MARCH3 | membrane-associated ring finger (C3HC4) 3 | NM_178450 | 1.639 | 0.387 |
| P4HA2 | prolyl 4-hydroxylase, alpha polypeptide II, transcript variant 1 | NM_004199 | 1.627 | <0.001 |
| NID2 | nidogen 2 (osteonidogen) | NM_007361 | 1.623 | <0.001 |
| XDH | xanthine dehydrogenase | NM_000379 | 1.616 | 0.387 |
| PLA1A | phospholipase A1 member A | NM_015900 | 1.558 | 2.075 |
| SRGN | serglycin | NM_002727 | 1.521 | <0.001 |
| ECE1 | endothelin converting enzyme 1 | NM_001397 | 1.477 | 0.595 |
| PLAUR | plasminogen activator, urokinase receptor | NM_002659 | 1.476 | 0.595 |
| ANPEP | alanyl (membrane) aminopeptidase | NM_001150 | 1.475 | 0.387 |
| CXCL6 | chemokine (C-X-C motif) ligand 6 (granulocyte chemotactic protein 2) | NM_002993 | 1.468 | 1.627 |
| PODXL | podocalyxin-like | NM_001018111 | 1.467 | 1.627 |
| PLAU | plasminogen activator, urokinase | NM_002658 | 1.460 | 0.387 |
| PLAT | plasminogen activator, tissue | NM_000930 | 1.451 | 1.627 |
| COL4A1 | collagen, type IV, alpha 1 | NM_001845 | 1.430 | 0.387 |
| VEGFC | vascular endothelial growth factor C | NM_005429 | 1.426 | 0.387 |
| BID | BH3 interacting domain death agonist | NM_197966 | 1.422 | 1.060 |
| GPRC5B | G protein-coupled receptor, family C, group 5, member B | NM_016235 | 1.420 | 0.595 |
| TFPI2 | tissue factor pathway inhibitor 2 | NM_006528 | 1.404 | 0.857 |
| ETS1 | v-ets erythroblastosis virus E26 oncogene homolog 1 (avian), transcript variant 2 | NM_005238 | 1.388 | 2.075 |
| CASP4 | caspase 4, apoptosis-related cysteine peptidase | NM_033306 | 1.355 | 3.152 |
| ACP2 | acid phosphatase 2, lysosomal | NM_001610 | 1.349 | 2.075 |
| DUSP10 | dual specificity phosphatase 10 | NM_007207 | 1.327 | 2.306 |
| KYNU | kynureninase (L-kynurenine hydrolase) | NM_003937 | 1.324 | 0.857 |
| EREG | epiregulin | NM_001432 | 1.312 | 3.186 |
| CFLAR | CASP8 and FADD-like apoptosis regulator | NM_003879 | 1.311 | 2.075 |
| STK17A | serine/threonine kinase 17a | NM_004760 | 1.302 | 2.075 |
